# A Donald F. Hunt Story (John’s Version)

**DOI:** 10.1016/j.mcpro.2024.100869

**Published:** 2024-10-24

**Authors:** John R. Yates

**Affiliations:** Departments of Molecular Medicine and Neurobiology, The Scripps Research Institute, LaJolla, California, USA

**Keywords:** Hunt laboratory in the 1980's, protein sequencing by tandem mass spectrometry, tandem mass spectrometry, standard test peptide-2, Protein Society, Methods in Protein Sequence Analysis

## Abstract

A personal narrative of my time in the Hunt laboratory and beyond is provided. The impact of the Hunt laboratory on the analysis of peptides and proteins by tandem mass spectrometry is described in the context of the time.

## Highlights

• Development of tandem mass spectrometry for sequencing peptides in the 1980’s.
• Efforts to raise awareness of tandem mass spectrometry for protein sequencing at conferences.

## Graphical Abstract

**Figure:**
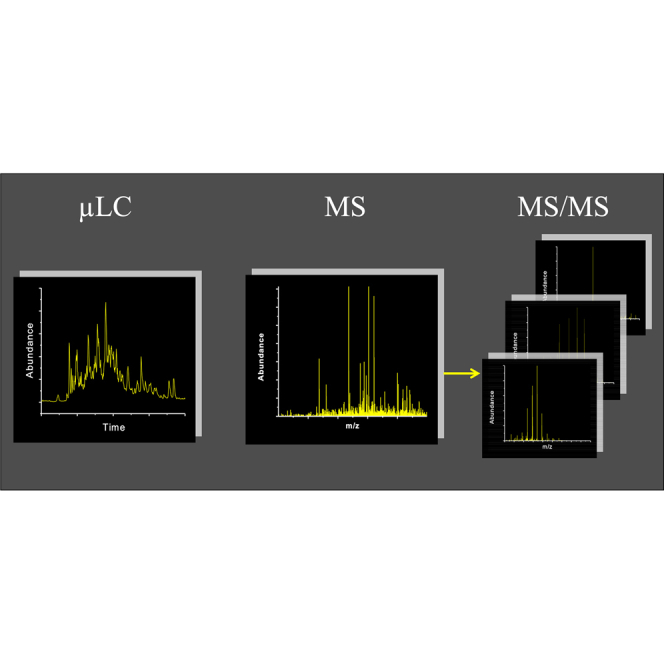

## In Brief

A personal narrative is provided on Donald Hunt’s development of tandem mass spectrometry methods for sequencing peptides.

As a post-doctoral fellow in Klaus Biemann’s laboratory (1968–1969), Donald F. Hunt (Don) was exposed to the early efforts to sequence peptides but did not work on a peptide sequencing project. Instead, Don worked on the analysis of dinucleotides by mass spectrometry (1) as a postdoctoral fellow, and when he began his independent career, he focused on the emerging field of chemical ionization mass spectrometry and ion-molecule reactions. After receiving tenure in 1974, Don left for a sabbatical in 1975 at Cambridge University in the laboratory of Dudley Williams, where he planned to revisit his early work on sequencing DNA by mass spectrometry. After settling in, Don met with Dudley to discuss his plans for the sabbatical. As he described his plan to develop a mass spectrometry approach to sequence DNA, Don said Dudley paused for a bit and then responded, “The Medical Research Council (MRC) has Wednesday evening lectures, and there is one this coming Wednesday you might find interesting”. On Wednesday Don rode over to the MRC on his bicycle to listen to Fred Sanger describe his *revolutionary* new DNA sequencing method (2), which immediately convinced Don to pivot his attention to peptide sequencing.

In the 1970s, the two powerhouses of protein sequencing by mass spectrometry were the Klaus Biemann Laboratory at MIT and the Howard Morris Laboratory at Imperial College in London (3, 4). The sequencing strategies employed in both labs involved the derivatization of peptides to make them volatile. The Biemann lab used a derivatization scheme to produce polyamino alcohols from peptides and the Morris lab employed the permethylation method developed by Das and Lederer that labeled amide nitrogens in the polypeptide backbone with methyl groups (5, 6). The polyamino alcohol method was distinguished by its use of di-, tri-, and tetrapeptides that were amenable to GC/MS after derivatization, while the permethylation approach used bigger peptides that were analyzed off a heated direct insertion probe.

At the 1978 ASMS meeting Richard Yost, a graduate student in Chris Enke’s laboratory, presented a lecture that reported the use of collision-induced dissociation (CID) on a triple quadrupole mass spectrometer (TSQ) (7). Yost had spent the summer working in James Morrison's laboratory at Latrobe University in Australia trying to demonstrate CID and to see how it compared to Mass-analyzed Ion Kinetic-Energy Spectrometry (MIKES) (8, 9). Don and Jeff Shabanowitz were in the audience and were excited by the possibilities of this instrument for peptide sequencing. On returning from the conference, Don instructed Jeff to dismantle the lab's three quadrupole mass spectrometers and use the parts to build a so-called triple quadrupole like the instrument Enke and Yost had described at ASMS, a task Jeff completed in 2 months. Jeff’s home-built instrument worked, and it allowed Don to describe the sequence analysis of permethylated peptides on a triple quadrupole mass spectrometer in time for the 1979 ASMS and ACS conferences (10, 11), with a peer-reviewed sequencing paper appearing in 1981 (11, 12). The Hunt laboratory’s early efforts for peptide analysis employed the permethylation method, but soon new methods arose that eliminated the need for derivatization.

Big leaps in mass spectrometry capability have been driven more by advances in ionization methods than by mass analyzers. Historically, the ability to create new types of ions or bigger ions has provided the motivation to develop mass analyzers to meet the new capabilities. Michael Barber discovered that bombarding a glycerol matrix with fast atoms would ionize underivatized peptides and many other non-volatile molecules (13, 14), but interestingly, the use of glycerol was not mentioned in the description of fast atom bombardment (FAB) in his first two papers (13, 15). Instead, the authors suggested that it was the fast atom beam that distinguished their method from bombardment with an ion beam (*e.g.* SIMS). Jeff noticed cluster ions every 92 Da and surmised the missing ingredient was glycerol. The experiment was tried, and Jeff was correct. Peptides were quickly analyzed by FAB/Liquid Secondary Ion Mass Spectrometry (LSIMS) on the triple quadrupole mass spectrometer to produce the seminal 1981 Hunt peptide sequencing paper published in *Analytical Chemistry* (16). Both ionization methods were reported in the paper and it turned out that bombardment with atoms or ions produced the same result.

By the time Don went on his second sabbatical in 1981, this time to Imperial College, the paradigm for peptide ionization was shifting to Mickey Barber's FAB technique and away from the derivatization techniques that were required for the earlier sequencing approaches (13). Howard Morris, as an early collaborator and colleague of Barber, got early access to the new ionization method (14), and soon FAB was being used on double-focusing magnetic sector instruments (the elite mass spectrometers of the day) to sequence peptides. FAB deposited enough internal energy to peptide ions to cause a low level of fragmentation (14). If the peptide applied to the probe tip was pure, then enough fragment ions could be obtained to read out the sequence. However, getting to a complete sequence was not always straightforward, and thus, methods had to be developed to help sort ambiguities in sequence calling (17). While Don was away, he would call Jeff in Virginia to tell him how to prepare the lab to focus on the new peptide sequencing methods he was learning at Imperial College. Upon returning from his sabbatical, Don, intended to focus solely on protein biochemistry.

In 1981, I was at the University of Maine taking a course on mass spectrometry taught by Robert Anderegg and we were given an assignment to profile the research of a prominent mass spectrometry laboratory. At the time, I was also working in Anderegg’s lab developing an ionization method using mixed gas charge exchange/chemical ionization to sequence peptides with the Biemann polyamino alcohol derivatization strategy on a Hewlett Packard GCMS. Because Anderegg had obtained his PhD in Biemann's laboratory, I was familiar with every paper published by the Biemann laboratory on polyamino alcohols (18), so I chose to research Don’s laboratory at the University of Virginia. The Hunt laboratory had recently published the paper using fast atom bombardment (FAB) with tandem mass spectrometry mentioned above, and even then I recognized that a method that did not require derivatization of peptides was the future of peptide sequencing by mass spectrometry (16). After doing the literature search required for my assignment, I was so excited that I wrote to Don to ask him what was new in the laboratory and about opportunities for furthering my graduate education there. To my amazement, Don wrote back in a handwritten note, even though at the time he was on sabbatical. He described his current research projects and encouraged me to apply for graduate school at the University of Virginia (UVA). Don’s handwritten note not only convinced me to continue to graduate school at UVA to focus on protein sequencing by tandem mass spectrometry, it also provided a remarkable display when I presented my project in class.

**Fig. 1:**
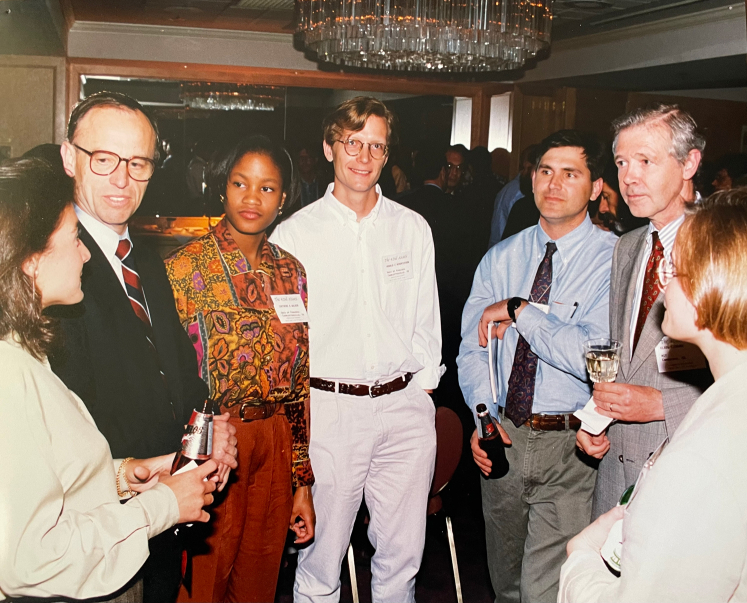
**Don at the 1994 42nd Annual ASMS conference**. From Left to right Dalene Kottmeier, Don, Carthene Bazemore-Walker, Ronald Hendrickson, John Yates, Klaus Biemann, and Pam Gulden. This picture originally appeared in the foreword of the special issue for Don’s 65th birthday in the *International Journal of Mass Spectrometry* 259, (2007) vii-xi.

The first time I met Don was at the 1983 ASMS opening reception in Boston. He immediately encouraged me to attend a talk about the external ionization source quadrupole FTMS built-in Bob McIver’s lab because Jeff was building one at UVA (19). Apparently, both Don and McIver had approached Finnigan MAT about building this instrument, but Don had contacted them about a week after they had agreed to work with McIver. Don’s enthusiasm convinced me that this instrument was going to be amazing, and evidently, others were equally enthused, since McIver’s talk at ASMS was standing room only (19). The concept behind the instrument was to place the ion source away from the ICR cell and to use quadrupoles to guide ions from the ion source into the ICR cell. By separating the ion source from the ICR cell by several feet, differential vacuum pumping would allow the use of methods such as FAB (and eventually ESI (20)), as it would be easier to keep the ICR cell at the 10^−9^ torr vacuum level. Additionally, the use of quadrupoles would allow the elimination of chemical noise, thus reducing the risk of overfilling the ICR cell with ions and creating a space-charging effect. The question McIver was addressing in his 1983 ASMS talk was whether you could inject ions through the fringing magnetic fields of a high field magnet and then trap the ions in the ICR cell, and the positive result described in his talk had encouraged Jeff and Don to move forward with building the instrument. Throughout the reception Don introduced me to other famous mass spectrometrists who stopped by to say hello while we were talking, and their energy and enthusiasm only fueled my eagerness to get to UVA (Fig. 1; shows Don interacting with students and the famous mass spectrometrist Klaus Biemann at the 1994 ASMS conference). At the end of the summer, I finished in Maine and headed to Charlottesville to claim a desk in the Hunt laboratory. Jeff had upgraded the laboratory’s triple quadrupole mass spectrometer with new RF power supplies to increase the mass range to 1800 Da and I was anxious to start research, but I had to wait until I had completed first year classes. It was common to find Don working in the lab at this time. He had bench space and would work on projects such as the analysis of polyglutamate side chains via the gamma carboxyl groups in folylpolyglutamates (21, 22), and he always wore his characteristic coat and tie when working at the bench. Since I was the only graduate student in the laboratory at this time, Don was the one who taught me how to reduce and alkylate proteins and then how to digest them with proteases. One of my first tasks in the laboratory was to install a 214 nm UV detector on the HPLC system Don had instructed Jeff to buy for peptide detection (this was my introduction to the frustrations of HPLC), and at the same time Jeff was starting to build the external ion source FTICR instrument (23, 24).

From circa 1983 to 1985, Charles Hauer and Patrick Griffin joined the Hunt lab as efforts to sequence proteins using tandem mass spectrometry were ramping up. I developed a collaboration with a fellow UVA chemistry graduate student Terry Zirino (Bruce Averill’s lab), who was studying the Iron Sulfur Center of purple acid phosphatase, a striking purple colored protein. This protein made a good target for protein sequencing, especially since Terry had to isolate large amounts of the protein for her studies. At some point, Don was approached by Keith Brew of the University of Miami and R. Michael Roberts from the University of Missouri about sequencing uteroferrin, which was added to my plate. Uteroferrin was also a bright purple color; interestingly, these two proteins were related and ended up having a high level of sequence homology (90%). Purple acid phosphatase was isolated from beef spleen while uteroferrin was isolated from porcine uterine fluids raising interesting questions about their functions (25, 26). At this time the lab was also sequencing apolipoprotein B which would become the center point for the 1986 seminal paper on “Protein Sequence Analysis by Tandem Mass Spectrometry” (27). In this paper, the details of our protein sequencing strategy were described, including the mechanisms of peptide fragmentation using CID. A complete sequence for Apo B was not included in the paper as it turned out the protein was greater than 500 kDa (28), which was a bit larger than the 50 kDa we expected it to be.

In the mid-1980s, tandem mass spectrometers based on double-focusing magnetic sectors started to appear (29, 30). Magnetic sector instruments were heavily pursued by many of the labs involved in protein sequencing by mass spectrometry because they were based on the most widely used mass analyzers, but they cost about three times as much as a triple quadrupole instrument. Among mass spectrometry protein sequencing labs, Don's lab was the highest profile (and by some reports, the only one) using the TSQ (31). As you might imagine, arguing in a grant proposal for the greater amount of money (∼$1.5 M in 1980s dollars) required to buy a double-focusing tandem mass spectrometer instrument necessitated stating why the less expensive TSQ option was not suitable. Those seeking funding for magnetic sector instruments argued so fiercely against the TSQ that NSF program officers drove to Charlottesville to meet with Don and ask if what was being said about the TSQ and protein sequencing in the grant proposals they were receiving was true or not. The attacks on the TSQ appeared in the popular science press as well (31). Don assured the NSF program officers that the criticisms were not true, and these attacks motivated him to offer a TSQ protein sequencing “boot camp” that taught the Hunt sequencing methods to over 400 people. With both camps dug in, this debate might have continued indefinitely if not for the introduction of electrospray ionization. Since magnetic sector instruments couldn’t scan fast enough to perform LC, they were incompatible with electrospray ionization, which almost immediately resulted in the universal adoption of the triple quadrupole mass spectrometer for peptide sequencing (32).

The early 1980s were also notable for the development of personal computing. I had taken a programming class before I arrived at UVA, and I expressed an interest in writing code for mass spectral data interpretation, so Don directed me to the UVA mainframe. However, at that time it was impossible to transfer mass spectrometry data to the mainframe, so it was not possible to do what I wanted to do. When the cost of personal computers started dropping rapidly, I bought my own PC so I could program in the laboratory. I soon noticed that Intelligenetics (the company that hosted GenBank) was selling a PC-based software package with protein analysis tools called PC/GENE. PC/GENE included versions of the protein and nucleic acid analysis programs that ran on mainframe computers. These programs had been ported onto the PC by Amos Bairoch (33), the creator of the Swiss-Prot database, which was also included in PC/Gene. PC/GENE introduced the lab to protein analysis software and introduced me to protein sequence databases (prior to PC/Gene my exposure to sequence “databases” was Margaret Dayhoff’s book “Atlas of Protein Sequences and Structures”) (34). Although PC/GENE’s data analysis tools were not explicitly needed for the ongoing studies in the lab, Don allowed us complete freedom to explore our own ideas. The unofficial motto in the lab was “do the experiment”. The insights I got from trying out the various programs included with PC/GENE got my mind racing about what could be done with computers, mass spectrometry data, and sequence databases. Even though we had PCs in the lab, getting data off the DEC PDP 11 computer that was used to operate our home-built triple quadrupole was still problematic. To reboot the computer, we had to toggle a collection of switches in the correct order, and data were stored on a 10 Mb magnetic removable data cartridge (about 18 inches in diameter!). The computer was not attached to the internet per se (there was ARPAnet and CSNET) and thus the only way to get the MS/MS data off the computer was to print out the m/z – intensity list for each spectrum. We had discussions with Finnigan MAT about this issue and they encouraged Don to upgrade the computer, but he was aware that a new triple quadrupole was coming and he didn’t want to spend the money. The Finnigan MAT TSQ70 was released in 1986, and the lab bought one a couple of years later. Around the same time, we felt that our protein sequencing work was making enough progress to reveal what we had been working on at a protein sequencing meeting.

In August 1986, the Methods in Protein Sequence Analysis (MPSA) meeting took place in Seattle. Both Don and I submitted abstracts, and I was selected to present a poster to report the acetylcholine receptor work being done in collaboration with Cynthia Moore and David Cafiso from the UVA chemistry department, while Don was selected for a talk (35). I have four distinct memories of this meeting: there was tremendous excitement around advances in protein sequencing methods, mass spectrometry was being featured among the traditional sequencing methods and gaining respect, the conference banquet was a wonderful roving buffet in the dorm cafeteria’s kitchen, and when Don and I were at the airport in Seattle waiting to board the flight home the gate agent handed us new boarding passes with an upgrade to first class. Flying cross country in first class was the icing on the cake to my first protein sequencing conference. I learned so much from this meeting that I was anxious to attend more, so after receiving a graduate fellowship from the Department of Chemistry, I asked Don if I could attend the Protein Society meeting in San Diego the next summer and he agreed. One of my goals for the meeting was to track down Reudi Aebersold and discuss 2D gel electrophoresis (2DGE) and his new electroblotting method (36). As a result of Reudi’s papers on the topic, Don was getting interested in how we could integrate 2DGE with mass spectrometry sequencing. I found Reudi at his poster in a dark corner of the poster hall and I was asking questions about increasing the loading of protein on a 2D gel as we still needed a nanomole of peptide for sequencing. I think I confused him as my questions showed a great lack of understanding of 2DGE, but Reudi patiently answered my questions, and I walked away impressed by this protein chemistry rockstar. Another exciting activity of the conference was the protein sequencing workshop run by Jean Rivier, who synthesized peptides in his laboratory at the Salk Institute. He would synthesize a peptide of unknown sequence and send it to all requesters, who would attempt to sequence it. Jean was known to add a twist to the sequence that was intended to keep people on their toes. People would go to the podium to present their results, but they were arranged so the people with incorrect answers went first, with the evening culminating in the presentation of the correct sequence. Jean was the master of ceremonies for the workshop as well as the architect of the polypeptide’s sequence. His goal for the workshop was to build the right answer with the early presenters showing how you could be distracted from the right answer. This workshop was particularly brutal to the participants, and their participation should be commended for a willingness to bare their scientific souls to a ballroom packed with their peers. When I returned to UVA, I was bubbling with excitement about how we should get samples for the next conference in 1988.

After attending the 1986 MPSA meeting in Seattle, Don was invited to speak at the 1988 conference in Berlin. At this conference, his talk about protein sequencing by tandem mass spectrometry and the new external ion source QFTMS instrument caught the attention of Leroy Hood from Caltech. Lee’s innovations with gas-phase/solid liquid protein sequencers almost a decade earlier had driven the protein chemistry field and were partly responsible for the formation of the MPSA and the Protein Society conferences. Now that many more protein sequences were becoming available, people could start answering questions about sequence/function and structure/function. Don and Lee had dinner to talk about mass spectrometry. Lee was not a novice when it came to mass spectrometry as he had collaborated with the Jet Propulsion Laboratory to use a mass spectrometer to detect and identify PTH amino acids (37). As the dinner was concluding Lee asked Don if he had any students graduating soon. Don mentioned me, thus planting the seeds of my future connection to Caltech.

By the time Don returned from the Berlin MPSA conference, the lab was busy characterizing symposium test peptide 3 (STP-3) for the upcoming Protein Society conference in San Diego. The peptide sequence that year was particularly tricky and even as Pat Griffin, Don and I arrived at the conference, we still didn't have a final answer. Don continued to look over data in his hotel room, racing to reach an answer. It turned out that the peptide consisted of two polypeptide chains: one attached through the alpha-amino group of a terminal Lys residue and one to the epsilon amino group. The first four amino acids of both chains were the same. Consequently, if you used Edman sequencing alone, you would get a signal that drops in half on the fifth cycle. Unless a mass spectrometer was used to measure the intact polypeptide’s molecular weight, you may not suspect it was two chains. Don got an answer and submitted it to Jean Rivier on a sheet of paper from a hotel room notepad. As this was our first time participating, we did not realize it was customary to provide supporting data. This lack of supporting data led Jean to assume that Don was guessing at the answer. Consequently, he did not assign Don a position in the presentation queue reserved for those presenting the correct solution. By the time Don got up to speak, no right answer had been presented, and Don was in his element, using overheads to show his interpretations of peptide tandem mass spectra, and culminating with the correct overall structure (38). Edman degradation was run on the sample late in the process but only to confirm the sequence (as mentioned above, Edman alone gets you the wrong answer). With the presentation of the correct sequence in the middle of the speaker’s queue, the excitement clearly had peaked. The other presenters simply confirmed Don's answer and then described how they got there, including Applied Biosystems (ABI), the leading manufacturer of protein sequencers who had shut down their protein sequencing group for a month to work on the peptide sequence. One clear outcome of the workshop was that tandem mass spectrometry had gained legitimacy as a protein sequencing technique in the eyes of the traditional protein sequencing community.

After returning from the conference, I packed up and headed to Caltech. In the 1980’s Caltech was the epicenter of a shift in the scientific paradigm led by the Hood laboratory. Lee Hood had been developing microchemical instruments to sequence and synthesize proteins and to sequence and synthesize DNA. With the development of the 4-color automated DNA sequencer, Lee Hood's group had become the “hotbed” of DNA sequencing and at the forefront of discussions for sequencing the human genome (39, 40, 41, 42). Many of the luminaries of the human Genome Project made the trek to Lee’s laboratory to discuss the project and see the DNA sequencer in action. ESI and MALDI had just been introduced and now mass spectrometry is being discussed as a potential advanced method for DNA sequencing for the human genome project. Lee had connections to the Department of Energy (DOE), so he, Tim Hunkapiller, and I made a visit to the Oak Ridge National Laboratory. Lee invited Don to attend the meeting as well. Of course, our goal was to convince the DOE to provide funding for mass spectrometry DNA sequencing, but the DOE was more interested in vetting their ideas on DNA sequencing methods (including mass spectrometry) to us (43). Even though I had left Don's lab we still maintained contact and he visited Caltech a few times while I was there. Through Don, I met Caltech’s Jack Beauchamp, and my little research group started meeting regularly with Jack and the people in his group who were interested in the mass spectrometry of proteins. Jack had built an FT-ICR system with the goal of using gas phase ion chemistry to effect an “Edman like” chemistry inside the ICR cell to sequence proteins (44).

One of my intentions in going to Caltech was to learn the process of 2D gel electrophoresis and to try to integrate the sequencing of proteins from the gels with tandem mass spectrometry. Ruedi Aebersold was at Caltech integrating 2DGE with Edman sequencing, but he left for the University of British Columbia just before I arrived. Lee had hired Michael Harrington to run the 2 DGE laboratory and he was much more focused on the 2DGE process (*e.g.* reproducibility) and software visualization of the gels then identifying proteins from the gels. At Caltech, we acquired a TSQ70 mass spectrometer, initially with a LSIMS ionization source, but eventually with an Analytica of Branford ESI source. Don’s lab had already gotten access to the ESI source and was testing the most sensitive ways to introduce peptides into the mass spectrometer. To make peptide sequencing methods more sensitive, Don’s lab began exploring capillary electrophoresis and then packed capillary columns for liquid chromatography (45, 46). Fortuitously, the lab that invented CE was not far away at the University of North Carolina and consequently, Don initiated a collaboration with Jim Jorgenson. Arthur Moseley was a graduate student in the Jorgenson lab and he traveled to UVA to work with Don and Jeff to test CE and then packed capillary columns (47). As my Finnigan TSQ70 at Caltech would soon be upgraded to ESI, I traveled back to UVA to learn these new methods. Jeff taught me how to pack capillary columns and showed me what they had learned about ESI on the TSQ70. Around this time, an emerging research area in immunology was the identification of peptides bound to major histocompatibility complexes (MHC), the system that presents antigens to CD8+ T-cells of the immune system. Don hypothesized that ESI LC-tandem mass spectrometry could be used to sequence the peptides presented by the MHC system (48).

I was still at Caltech in 1990 when Don was contacted by Wilhelm Ansorge who was trying to fill a group leader’s position at the EMBL for the peptide/protein chemistry group. Rather than go the traditional route of Edman sequencing, Ansorge wisely looked at mass spectrometry as an alternative. Don gave him my name and after interviewing, I was offered the position. It was a hard decision, but I turned the position down as I calculated it would be difficult to return to the USA with a tenured faculty position no matter how well I did at the EMBL. So, I was still interviewing for USA faculty positions in 1991 at the same time Lee Hood was attempting to relocate his laboratory to another university. The University of Washington (UW) was interested in recruiting Lee but realized it would take substantial funding to move him. UW introduced Lee to Bill Gates, who had already made a fortune at Microsoft but had not yet begun funding philanthropic projects. Lee and Gates hit it off and Gates provided $12 million for Lee to start a new department at UW (49). I was offered a tenure-track position in the new department and chose to join UW over an offer from the chemistry department at the University of North Carolina. I was in touch with Don during this process and he provided wise counsel as I struggled to decide. It was a difficult decision, but I reasoned that being in a biology department with a strong emphasis on the genome project could present a significant advantage. My exposure to the genome project while at Caltech had a profound effect on how I was thinking about mass spectrometry and protein sequencing.

One of my first research efforts while starting my laboratory at UW was database searching using mass spectrometry data. At Caltech, I had been working on mass mapping using LCMS data. While working on the paper I had to think about the method’s limitations and noted in the conclusions that if a match was ambiguous, you could collect a tandem mass spectrum to resolve the ambiguity (50). In the lab we were sequencing MHC Class II peptides and after several frustrating incidents of fully sequencing a peptide, only to learn through a BLAST search that the peptide sequence was known, we modified our approach to partially sequence a peptide to obtain a sequence of four or five amino acids which would then be used in a BLAST search (51, 52, 53). When we got the search results, we could align them to the spectrum. BLAST searches were performed using an e-mail server which required sequences to be emailed to the server. While sitting at the computer waiting for a search to return, I thought, “why can't I just use a tandem mass spectrum to match it to a sequence in the database?”. After testing the idea with a small database I constructed and an MS/MS of a peptide, I hired Jimmy Eng to work on the problem. Within a couple of months SEQUEST was created. However, creating the algorithm proved to be easier than getting the paper published.

The first version of the SEQUEST paper was sent to Fred McLafferty for consideration as an article in *PNAS.* It was reviewed but rejected. I had thought McLafferty would appreciate the method as it was similar in concept to the mass spectral library searching method he had developed decades earlier. McLafferty’s advice was to revise the paper and send it somewhere else, but have the editor contact him and he would reveal who the reviewers were and that would speed up the process. This ended up being a big mistake as it appeared that Russell Doolittle, the new editor at *Protein Science*, only received one reviewer's name or only had used one of the reviewers, and that reviewer wrote strongly negative reviews, leading to two more rejections. This person revealed himself to a friend some years later and took some pride in rejecting the paper. The last rejection came just before the American Society for Mass Spectrometry Conference, consequently, I tracked down Don at the conference to get advice. Don said, “Do you have a copy of the paper?” (I had sent Don earlier drafts of the paper). I handed him a copy and he said he was going looking for Michael Gross, the editor of the *Journal of the American Society for Mass Spectrometry*. Don came back and said he handed Mike the paper and told him, “you need to publish this paper”. After ASMS, Mike wrote and said to send him the previous reviews with responses. He got back to me and said this isn’t as bad as the reviewer claimed. He made some editorial suggestions for the paper and said that if I fixed those issues he would publish the paper (54). In the end, the paper was published, and it remains the most highly cited paper in the journal to this day. This story illustrates the fundamental role and impact a great mentor can have even after you have left their laboratory. I have tried to model this behavior, (both Don’s and Mike’s) in my role as a mentor and editor, bending over backward to help junior faculty get their papers published and with their research efforts.

The strengths of the tandem mass spectrometry approach to peptide sequencing are evident. The method is unbiased in the sense that fragment ion signals are not compared to a standard to obtain the amino acid sequence. The readouts are fragment ions whose differences in m/z provide the weight of the amino acid at that location in the sequence, so it is evident if the peptide is modified or unusual. In 1987 Michel and Bennett showed phosphopeptides could be enriched using Immobilized Metal Affinity Chromatography (IMAC) for Edman sequencing (55). Shortly thereafter Don collaborated with Michel and Bennett to sequence Fe^3+^-IMAC enriched phosphopeptides by tandem mass spectrometry (55, 56, 57). By using the phosphopeptide sequencing capabilities of the tandem mass spectrometer important biological discoveries were made in the Hunt laboratory (58, 59). Mixture analysis capability of tandem mass spectrometers was a strength of the technique in analytical chemistry and this was realized early in the use of tandem mass spectrometry for peptide sequencing. In Hunt’s 1986 paper, this advantage is described, “Another important feature of the tandem mass spectrometry method is that it does not require that the sample be purified to a high degree of homogeneity (27). The approach works well as long as the target protein is a major component of the protein mixture.” A previous Hunt laboratory study in which the protein being sequenced was not homogeneous (60) is cited in the PNAS paper. Because of this capability, we showed SEQUEST could be used to identify peptides/proteins in digested complex protein mixtures (*e.g. Saccharomyces cerevisiae* proteome) using tandem mass spectra. In 2002, Don and colleagues took this one step further and combined the IMAC enrichment of phosphopeptides to undertake a large-scale analysis of the phosphoproteome of *S. cerevisiae* (61) establishing the ability to perform large-scale analyses of modifications in a proteome. The combination of phosphopeptide sequencing methods with enrichment strategies remains a standard approach used to identify 1000s of modified peptides in proteomic experiments.

**Fig. 2:**
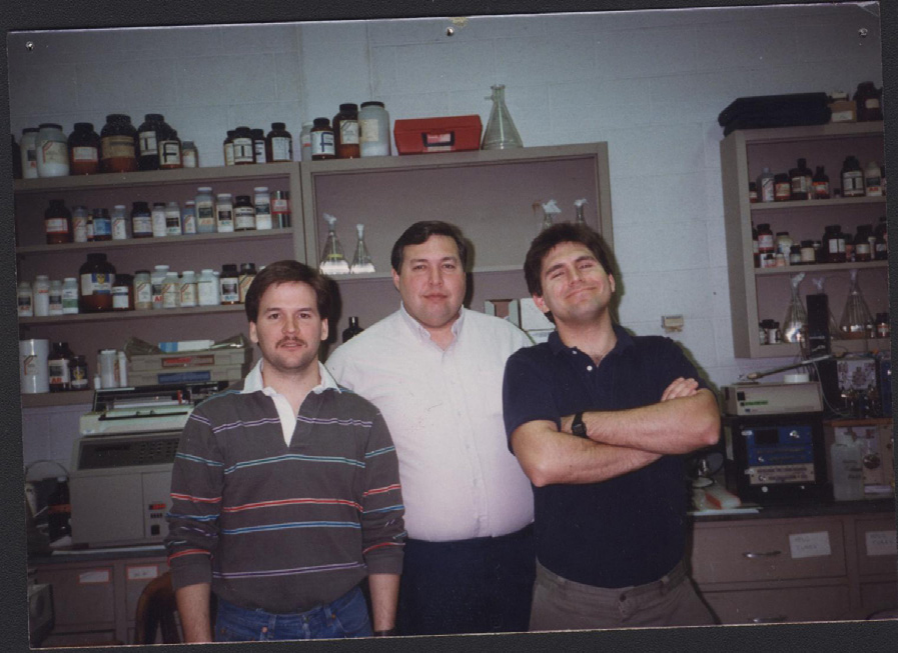
**The “three amigos” circa 1985; Patrick Griffin (*left*), Jeffrey Shabanowitz (*center*), and John Yates (*right*) standing in front of the Applied Biosystems Model 130 HPLC pump in the back of the Hunt lab**.

**Fig. 3:**
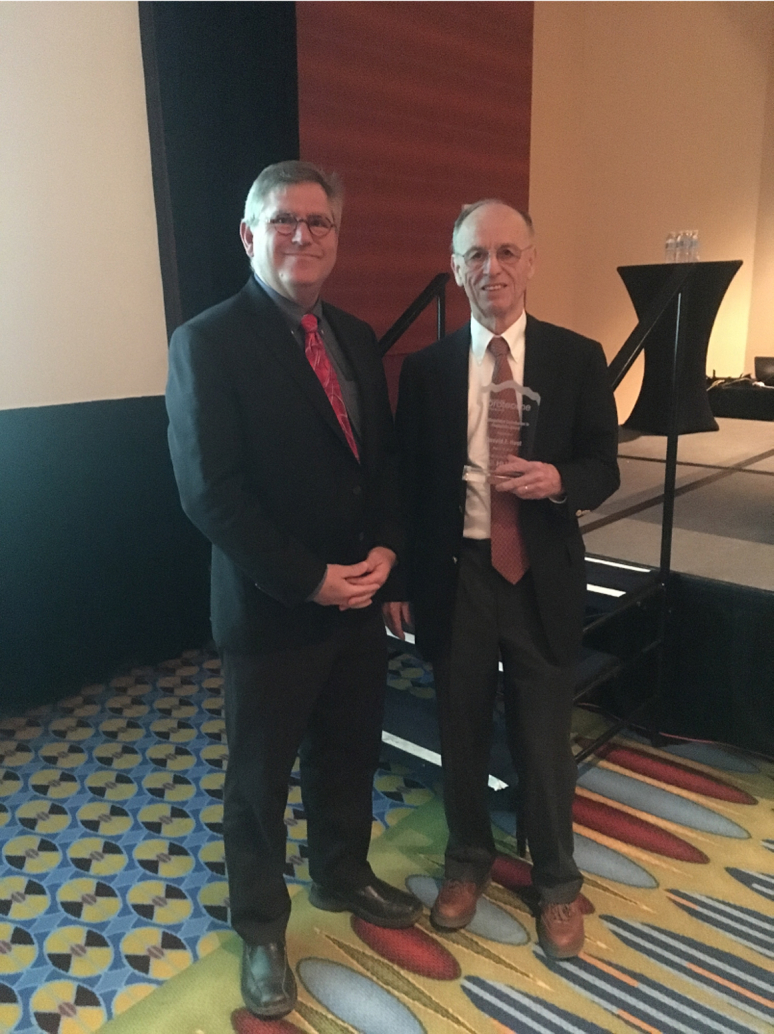
**Donald Hunt with the author after receiving the inaugural 2018 Distinguished Achievement Award in Proteomics which was subsequently named for Don; the Donald F. Hunt Distinguished Achievement Award in Proteomics**.

I have often said that my time in Don’s lab was some of the best times of my life. It was an intellectually immersive time that I shared with the other lab members, in particular Patrick Griffin and our lab teacher and mentor Jeff Shabanowitz (Fig. 2). As we pursued Don’s vision of protein sequencing by tandem mass spectrometry, we knew we had a lot of ground to make up to catch the Edman Degradation instrument and that we were the underdogs. Enhancing the experience was Don’s enthusiasm for new ideas, and for trying new experiments to push progress along. There was often the 4 PM experiment when Don came rushing into the laboratory asking Jeff to try an experiment he had just thought of that was usually centered around the new QFTMS instrument. If you are lucky, there are key people in your life that are both inspirational and transformational. Don was undeniably just that for me. His mentoring and confidence in me helped set me on my course and his influence is felt in the students and postdocs I have mentored in the 35 years since I had the privilege of working with him (Fig. 3).

## Data Availability

All supporting data are provided within the manuscript, supplementary data and supplementary tables.

## Conflict of interest

The authors declare that they have no conflicts of interest with the contents of this article.
